# The interrater and test–retest reliability of 3 modalities of quantitative sensory testing in healthy adults and people with chronic low back pain or rheumatoid arthritis

**DOI:** 10.1097/PR9.0000000000001102

**Published:** 2023-10-10

**Authors:** Sophia M. Brady, Vasileios Georgopoulos, Jet J.C.S. Veldhuijzen van Zanten, Joan L. Duda, George S. Metsios, George D. Kitas, Sally A.M. Fenton, David A. Walsh, Daniel F. McWilliams

**Affiliations:** a School of Sport, Exercise and Rehabilitation Sciences, University of Birmingham, Birmingham, United Kingdom; b Rheumatology Department, Dudley Group NHS Foundation Trust, Dudley, United Kingdom; c Medical Research Council-Versus Arthritis Centre for Musculoskeletal Ageing, University of Birmingham, Birmingham, United Kingdom; d Pain Centre Versus Arthritis, NIHR Nottingham Biomedical Research Centre, Advanced Pain Discovery Platform & Academic Rheumatology, School of Medicine, University of Nottingham, Nottingham, United Kingdom; e Sherwood Forest Hospitals NHS Foundation Trust, Nottingham, United Kingdom; f Department of Nutrition and Dietetics, School of Physical Education, Sport Science and Dietetics, University of Thessaly, Thessaly, Greece; g Faculty of Education, Health and Wellbeing, University of Wolverhampton, Wolverhampton, United Kingdom

**Keywords:** Quantitative sensory testing, Rheumatoid arthritis, Low back pain, Reliability

## Abstract

**Introduction:**

Quantitative Sensory Testing (QST) modalities used to assess central pain mechanisms require different protocols in people with different musculoskeletal conditions.

**Objectives:**

We aimed to explore the possible effects of musculoskeletal diagnosis and test site on QST interrater and test–retest reliability.

**Methods:**

The study included participants with rheumatoid arthritis (RA, n = 18; QST conducted on lower leg) and low back pain (LBP, n = 25; QST conducted on forearm), plus 45 healthy control participants (n = 20 QST on lower leg and n = 25 QST on forearm). Test–retest reliability was assessed from QST conducted 1 to 3 weeks apart. Quantitative sensory testing modalities used were pressure pain detection threshold (PPT) at a site distant to tissue pathology, temporal summation (TS), and conditioned pain modulation (CPM). Temporal summation was calculated as difference or ratio of single and repeated punctate stimuli and unconditioned thresholds for CPM used single or mean of multiple PPTs. Intraclass correlation coefficients (ICCs) were compared between different subgroups.

**Results:**

High to very high reliability was found for all assessments of PPT and TS across anatomical sites (lower leg and forearm) and participants (healthy, RA, and LBP) (ICC ≥ 0.77 for PPT and ICC ≥ 0.76 for TS). Reliability was higher when TS was calculated as a difference rather than a ratio. Conditioned pain modulation showed no to moderate reliability (ICC = 0.01–0.64) that was similar between leg or forearm, and between healthy people and those with RA or LBP.

**Conclusion:**

PPT and TS are transferable tools to quantify pain sensitivity at different testing sites in different musculoskeletal diagnoses. Low apparent reliability of CPM protocols might indicate minute-to-minute dynamic pain modulation.

## Abstract 2

Supplemental Digital Content is Available in the Text.

Quantitative sensory testing is a reliable tool to assess central pain mechanisms in healthy participants and people with rheumatoid arthritis and low back pain.

## 1. Introduction

Pain is a multidimensional sensory experience. Reliable measurement is essential to pain mechanism research in humans,^1^ and interest is growing in the field of musculoskeletal research about central aspects of pain.^24,53^ Quantitative sensory testing (QST) is an umbrella term for noninvasive psychophysical tissue-stimulation tests that provide information about pain processing^1,10^ and peripheral or central sensitisation of nociceptive signalling from sites that are at or remote from tissue injury.^1,38^ Quantitative sensory testing has been used to explore underlying pain mechanisms, such as sensitivity and dysregulation of ascending and descending pathways, in people with musculoskeletal conditions,^13,47^ including those with quite different aetiologies such as rheumatoid arthritis (RA) or low back pain (LBP).^20,25,54^

There are multiple different QST modalities designed to assess pain and other sensations and provide mechanistic insights. A battery of QST modalities could include measurements of thresholds for detection of cold, warm, or mechanical stimuli, for perceiving stimuli as painful, and measurements of pain intensity.^52^ One way in which QST can be categorised is into “static” (eg, pressure pain detection threshold [PPT] or tolerance thresholds) or “dynamic” (eg, temporal summation [TS] and conditioned pain modulation [CPM]) where changes in perception of a standardised stimulus are measured with repeated stimulus application or in the presence of a heteropic (conditioning) stimulus. Protocols can include measurements using a pressure algometer to assess PPT or weighted punctate probe and painful conditioning stimuli such as ischaemic arm pain induced by inflation of a blood pressure cuff.^37^ In combination, these assessments are valuable in providing insight into processing of nociceptive signalling within the central nervous system.^56^ Researchers often perform assessments at painful index sites, or sites of pathology, for QST, which will therefore be influenced by a mixture of peripheral and central pain mechanisms, plus possibly from local pain at the time. Selection of sites distant from the index site is recommended for assessment of central aspects of pain.^55^

Use of QST in research presumes measurement of a meaningful characteristic with tools that are reproducible, irrespective of the assessor (interrater reliability), and when the test is repeated (test–retest reliability).^22^ Quantitative sensory testing reliability has been reported in healthy people^8,19,34^ and people with neuropathic or osteoarthritis pain.^38,55^ In RA, Lee et al.^29^ reported a range of interrater reliabilities for PPT, TS, and CPM. In LBP, Paungmali et al.^46^ examined test–retest reliability of PPT on the primary region to which clinical pain was attributed, thought to be largely influenced by peripheral sensitisation. Central mechanisms might determine pain from stimuli at sites distant from pathology, with less influence from peripheral sensitisation than from stimuli local to the pathology.^55^ Sites of pathology differ between different musculoskeletal conditions, and therefore, QST might be undertaken at different body sites, and protocols are adapted for specific diagnoses. There is potential for shared methodologies when examining central pain hypersensitivity.^15,16^ Tibialis anterior muscle might be a suitable site in people with RA, away from affected joints, whereas nerve root involvement in LBP might necessitate an alternative test site such as brachioradialis muscle. Results of QST can vary between body sites, possibly because of differences in innervation of subcutaneous tissues or depth of overlying soft tissue.^23,28^ Furthermore, other clinical features such as disease activity, mental health, or disease flares might influence QST outcomes.^25,55^ However, much less reliability data are available comparing QST protocols tailored for assessing central aspects of pain in multiple different clinical populations.^17,29,46,58^

There are many QST protocols in use at sites remote from the index site of pain and limited standardisation in reporting of between- and within-study reliability in people with RA and LBP.^14,16,29,46,51^ Reliability assessment quantifies the reproducibility of a measure, enabling interpretation of variation because of experimental manipulations or pathological conditions that are greater than the expected variation because of random and systematic factors. One study reported that QST measurements can be very stable over a period of 10 weeks.^41^ We posit that a standardised, reliable, QST protocol, which could be used at pain-free sites across multiple musculoskeletal conditions, would enable collection of more harmonious data.^38^ Primary aims of this study were to establish the validity and evaluate test–retest and interrater reliability of PPT, TS, and CPM protocols that had been adapted for use at different remote testing sites in different clinical populations. Secondary aims were to define optimally reliable calculation methods for calculation of TS and CPM.

## 2. Methods

### 2.1. Participants

People with RA were recruited in person from outpatient clinics at Russells Hall Hospital, Dudley, United Kingdom. In addition, people living with LBP were also recruited in person by a member of the clinical care team,^15^ whereas people living with RA were recruited via telephone. Both population with a lived experience of LBP or RA were recruited from a list of participants who had already agreed to participate in research at the Universities of Nottingham, United Kingdom (LBP^forearm^) or Birmingham, United Kingdom (RA^leg^) and had consented to be recontacted (RA: 27 contacted and 18 participated, LBP: 40 were contacted and 25 participated). Healthy individuals (Healthy^leg^ for comparison with RA^leg^ and Healthy^forearm^ for comparison with LBP^forearm^) were recruited to assess and compare the reliability of QST modalities when conducted at different testing sites. Healthy individuals affiliated with the School of Sport, Exercise and Rehabilitation Sciences, University of Birmingham or Academic Rheumatology, University of Nottingham, were approached in person and invited to participate (Nottingham: 28 contacted and 25 participated; Birmingham: 22 contacted and 20 participated). Written informed consent was obtained from all individuals before participation.

Inclusion criteria for healthy individuals were as follows: adults (≥18 year old), having no acute or chronic pain, and understanding English. Exclusion criteria were as follows: diagnosed with another acute or chronic painful condition, current participation in a rehabilitation program, or pregnancy. Inclusion criteria for patient participants were as follows: adults, physician diagnosis of RA (RA^leg^ group) or chronic LBP (LBP^forearm^ group), and understanding English. People were excluded if unable to give informed consent because of cognitive impairment, history of comorbidities causing greater current disability than their RA or LBP (such as cancer or diabetic neuropathies), or pregnancy.

Favourable ethical opinions were granted from the University of Birmingham Ethics Committee, Black Country Regional Ethics Committee of the Health Research Authority (16/WM/0371), Faculty of Medicine & Health Sciences Research Ethics Committee of the University of Nottingham (264-1803) and East Midlands—Nottingham 1 Research Ethics Committee of the Health Research Authority (18/EM/0049).

### 2.2. Study procedures

Individuals with RA (RA^leg^) visited Russells Hall Hospital, and individuals with LBP (LBP^forearm^) visited King's Mill Hospital (Sutton-in-Ashfield). Healthy participants visited the School of Sport, Exercise and Rehabilitation Sciences, University of Birmingham (Healthy^leg^) or Academic Rheumatology, University of Nottingham (Healthy^forearm^) to take part. For test–retest reliability analysis, each participant (RA^leg^, LBP^forearm^, and Healthy^forearm^) undertook 2 QST sessions (baseline/follow-up) separated by 1 to 3 weeks. These timeframes were considered appropriate periods between sessions to reduce the risk of potential recall bias for research participants^38^ but are shorter than those generally used for clinical follow-up of chronic pain patients. Baseline and follow-up examinations were performed by the same researcher (rater 1 [SB] for Healthy^leg^ and RA^leg^ participants, rater 3 [VG] for LBP^forearm^ and Healthy^forearm^ participants). All participants completed the protocol in full, with a mean baseline to follow-up period of 8 days for Healthy^forearm^ and LBP^forearm^, 13 days for Healthy^leg^ and 12 days for RA^leg^ participants. Raters were fully trained on how to conduct the QST modalities, and procedures were standardised.

For interrater reliability, the same second rater was included in the baseline sessions for all healthy volunteers (Healthy^leg^ and Healthy^forearm^) (rater 2: DM).

### 2.3. Quantitative sensory testing

The QST protocol comprised both “static” (PPT) and “dynamic” (TS and CPM) modalities^2,51,63^ to measure sensitivity to mechanical stimuli (PPT), effectiveness of descending modulation (CPM), or degree of spinal sensitisation (TS). For Healthy^leg^ and RA^leg^ participants, testing was on the dominant leg at the tibialis anterior muscle (5 cm distal to tibial tuberosity and knee joint) for PPT and CPM modalities, and 5 cm above the patella on the skin above the rectus femoris for TS. For Healthy^forearm^ and LBP^forearm^ participants, test sites on both brachioradialis were 5 cm distal from the lateral epicondyle, corresponding with the body of the muscle. The distribution of testing sites was selected to attempt to facilitate comparisons between studies of different medical conditions with different patterns and index sites of pain. All participants were positioned on a lying position on a couch, with the upper body propped up.

Pressure pain detection threshold: For measuring PPT, an electronic hand-held algometer (Medoc-AlgoMed, Israel) was used. Increasing pressure with a 1-cm^2^ rubber probe of the algometer was applied on the dominant tibialis anterior (Healthy^leg^ and RA^leg^ participants) or nondominant brachioradialis (Healthy^forearm^ and LBP^forearm^ participants) at a rate of 50 kPa/s.^51^ Each participant was asked to press a button using their dominant hand as soon as the sensation of pressure started to become painful.^51^

Temporal summation: The TS was assessed by repeated application of a stimulus using the retractable blunt needle of a specially manufactured pen (256 mN Pinprick; MRC-Systems, Heidelberg, Germany). The participants maintained a relaxed position, and a single stimulus with the blunt needle was applied to skin above their dominant rectus femoris (Healthy^leg^ and RA^leg^ participants) or dominant brachioradialis (Healthy^forearm^ and LBP^forearm^ participants), followed by 10 repetitive stimuli at a rate of 1/s.^1^ After the single stimulus, each participant was asked to rate the experienced intensity of pain/sharpness on a 0 to 10 numerical rating scale (NRS) (Healthy^leg^ and RA^leg^ participants) or a 10-cm visual analogue scale (VAS) (Healthy^forearm^ and LBP^forearm^ participants) where the lowest and the highest extremes signified no pain/sharpness and worst imaginable pain/sharpness, respectively. After the 10 stimuli, they were asked to rate the average intensity of pain or sharpness on the same scales. Data were collected from 2 repeats of each of the single and 10 stimuli, with at least 2 minutes between repetitions.

Conditioned pain modulation: An unconditioned PPT measurement was first assessed in an identical way as described for PPT testing (PPT^Unc^). Then conditioned PPT was then assessed by repeating PPT testing while ischaemic pain (conditioning stimulus) was induced in their nondominant (Healthy^leg^ and RA^leg^) or dominant (Healthy^forearm^ and LBP^forearm^) arm by application of a 15-cm-wide blood pressure cuff (PPT^Con^). The cuff was inflated above systolic pressure to occlude arterial blood flow to the arm, and participants repeatedly squeezed a small foam ball. Once pain reached 4/10 rating, the conditioned PPT was performed on the dominant tibialis anterior (Healthy^leg^ and RA^leg^) or nondominant brachioradialis (Healthy^forearm^ and LBP^forearm^), followed by immediate release of the pressure cuff.

### 2.4. Data analysis

Sample size calculations for this study were performed with type I and type II errors as 0.05 to 0.20, respectively.^59^ With a minimally accepted reliability of ρ = 0.4 or ρ = 0.5 and an expected reliability of ρ = 0.8, the minimum sample sizes were calculated to be 19 or 22 subjects, respectively.^3,33^

Pain detection threshold was taken as the arithmetic mean of 3 replicate measurements (PPT^mean^), with lower PPT indicating greater pain sensitivity. Temporal summation was calculated as the difference (pain rating of the single stimulus subtracted from rating of average pain experienced during the 10 subsequent stimuli, TS^WUD^). The mean of the 2 TS^WUD^ values was used for analysis. The wind-up ratio, TS^WUR^, was calculated as the average pain during the 10 stimuli divided by pain rating of single stimulus. A larger positive value of TS^WUD^/TS^WUR^ indicated greater sensitivity. Conditioned pain modulation was taken to be the single conditioned PPT measurement (PPT^Con^) minus the arithmetic mean of the replicated unconditioned PPT measurements (PPT^mean^) (CPM^PPT-mean^).^62,63^ Conditioned pain modulation was also calculated using the single conditioned PPT measurement (PPT^Con^) minus the interim unconditioned PPT measurement (single measure taken immediately before the conditioning stimulus, PPT^Unc^) (CPM^Unc^). In both calculation methods (CPM^PPT-mean^ and CPM^Unc^), a lower value indicated higher sensitivity.^35^

TS^WUD^ and TS^WUR^ distributions in Healthy^leg^ participants, TS^WUR^ in RA^leg^ participants, and all PPT, TS, and CPM variables in Healthy^forearm^ and LBP^forearm^ participants all significantly differed from normality (positively skewed). TS^WUR^ and TS^WUD^ variables in all participants were logarithmically transformed to ensure data fit normality assumptions in subsequent analyses. In cases of values of zero, 0.1 was added as a small constant to allow logarithmic transformation.^4^ Where appropriate, nonparametric statistical tests were used. To assess differences between variables, paired samples *t* tests (normal data) and Wilcoxon signed rank tests (nonnormal data) were performed. Unpaired *t* tests (normal data) and Mann–Whitney *U* tests (nonnormal data) were conducted to examine for differences between participant groups and differences in modalities between sexes. To assess associations between QST modalities and between each modality with age, Spearman correlation coefficient tests were conducted.

The test–retest reliability and interrater reliability of the PPT, TS and CPM modalities were established using methods that focused on the measurement of reliability.^5,32,38,55,60^ A 2-way random effects absolute agreement model for single measures was used to measure the interrater reliability and the test-retest reliability. The intraclass correlation coefficient (ICC) with 95% confidence intervals (95% CI) were reported. For interpretation, ICC of <0.5 = low reliability, 0.50 to 0.74 = moderate reliability, 0.75 to 0.9 = high reliability, and >0.90 = very high reliability.^50^ Further analysis involved comparing differences between separate ICCs by testing differences in variances using F-distributions.^12^

Bland–Altman analysis was conducted to give a visual representation of the data and allow identification of systematic differences between measurements for each outcome (data not transformed). Plots show the mean difference (mean bias) between the 2 measurements and 95% upper and lower limits of agreement (LoA; each with 95% CI).^32^ An even distribution across the Bland–Altman plots indicated no evidence of systematic bias.^6^

Data were analysed using IBM SPSS V26 and R (V3.4.2), and *P* ≤ 0.05 indicated statistical significance.

## 3. Results

Participant characteristics are displayed in Table 1. Study groups comprised 25 Healthy^forearm^, 25 LBP^forearm^, 18 RA^leg^, and 20 Healthy^leg^ participants. Healthy participants were significantly younger than disease groups (RA^leg^ and LBP^forearm^: *P* < 0.001), with no differences between sexes (RA^leg^: *P* = 0.16, LBP^forearm^: *P* = 0.57).

**Table 1:** Characteristics of the participants.

	Healthy^leg^	RA^leg^	Healthy^forearm^	LBP^forearm^
N	20	18	25	25
Age median (IQR) years	26 (23–32)*	58 (55–65)*	31 (28–46)†	57 (48–65)†
Sex (n = female (%))	10 (50.0)	13 (72.2)	15 (60)	17 (68)

* Significant difference between Healthy^leg^ and RA^leg^ participants in demographic data, determined by independent samples *t* tests (age) and χ^2^ tests (sex).

† Significant difference between Healthy^forearm^ and LBP^forearm^ participants in demographic data, determined by Mann–Whitney *U* tests (age) and χ^2^ tests (sex) (*P* < 0.05).

IQR, interquartile range; LBP, participants with low back pain; QST, quantitative sensory testing; RA, participants with rheumatoid arthritis.

**Figure 1.**
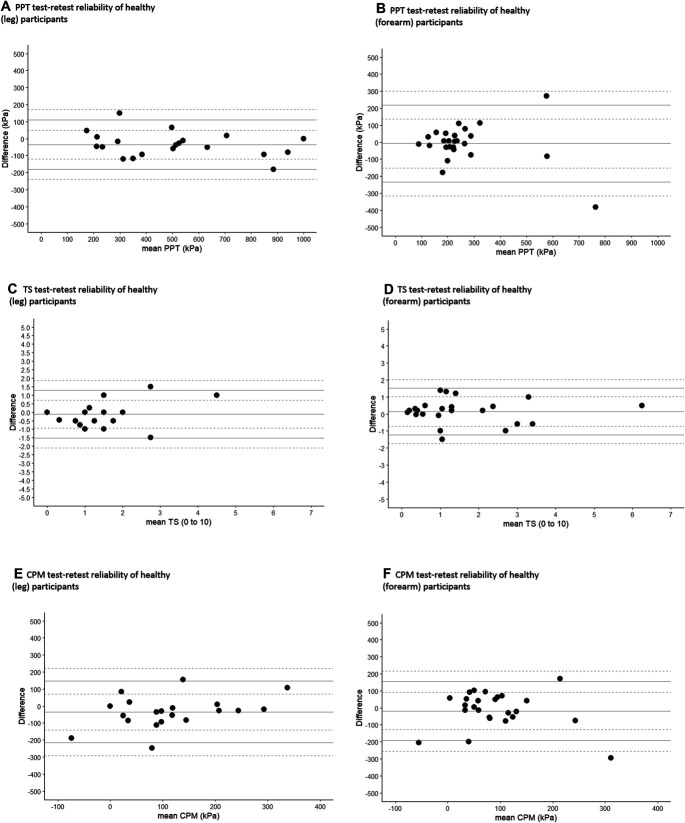
(A–F) Test–retest Bland–Altman plots for all QST modalities across healthy populations. CPM^PPT-mean^, conditioned pain modulation where the mean of the 3 PPT measurements was used as an unconditioned stimulus; LoA, limit of agreement; PPT, pressure pain threshold; TS^WUD^, temporal summation calculated as a difference.

**Figure 2.**
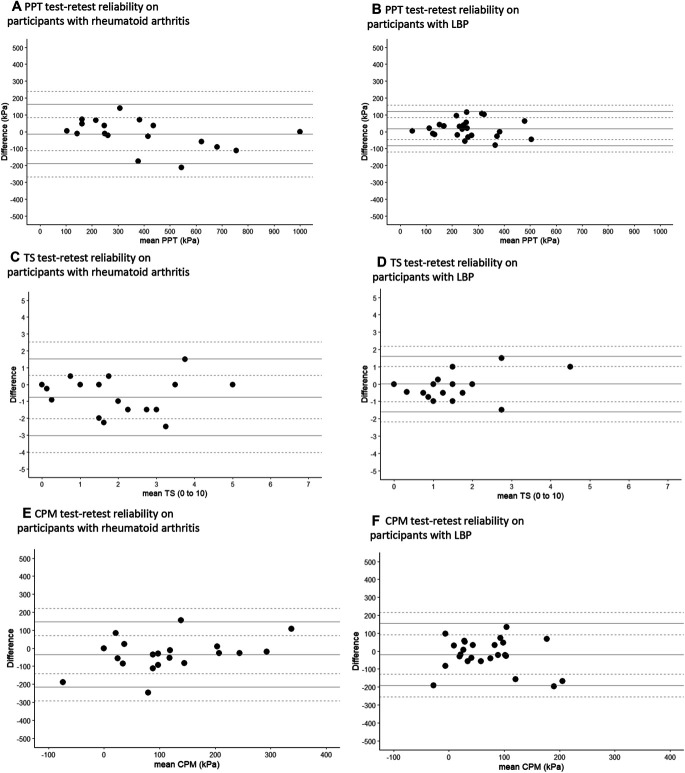
(A–F) Test–retest Bland–Altman plots for all QST modalities across RA^leg^ and LBP^forearm^ populations. CPM^PPT-mean^, conditioned pain modulation where the mean of the 3 PPT measurements was used as an unconditioned stimulus; LBP, participants with low back pain; LoA, limit of agreement; PPT, pressure pain threshold; RA, participants with rheumatoid arthritis; TS^WUD^, temporal summation calculated as a difference.

**Figure 3.**
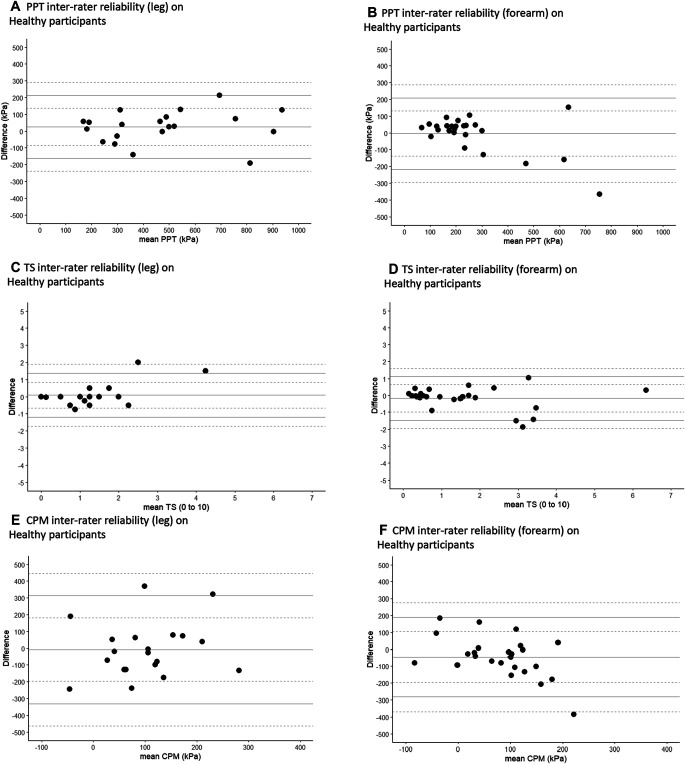
(A–F) Interrater Bland–Altman plots for all QST modalities across healthy populations. CPM^PPT-mean^, conditioned pain modulation where the mean of the 3 PPT measurements was used as an unconditioned stimulus; LoA, limit of agreement; PPT, pressure pain threshold; TS^WUD^, temporal summation calculated as a difference.

At baseline, PPT measurements were similar between replicates (Table 2). The interrater and test–retest ICCs for PPT were between 0.77 and 0.95, classified as high to very high at the forearm and very high reliability at the lower leg (Table 3). Bland–Altman plots did not show systematic variability of PPT between measurements (Figs. 1a, b, 2a, b, 3a, b, Supplementary Table 1, available at http://links.lww.com/PR9/A208). The ICCs for interrater reliability were statistically similar between lower leg and forearm, except that the test–retest ICC for PPT was significantly higher in Healthy^leg^ population (ICC = 0.95) compared with the Healthy^forearm^ population (ICC = 0.77, F [19,24] = 4.6, *P* < 0.001).

**Table 2:** Quantitative sensory testing measurements of all participants at baseline and follow-up.

Quantitative sensory testing	Baseline			Follow-up	
	Healthy^leg^		RA^leg^	Healthy^leg^	RA^leg^
	Rater 1	Rater 2	Rater 1	Rater 1	Rater 1
Lower leg					
PPT (kPa)	483.0 (259.9–689.3)	441.8 (281.8–567.5)	333.0 (232.7 to 488.6)	498.5 (269.2–688.0)	310.2 (173.1 to 650.2)
TS^WUD^ (−10 to 10)	1.0 (0.5–1.9)	1.3 (0.6–1.5)	1.5 (0.5 to 2.1)*	1.1 (1.0–2.0)†	2.6 (0.9 to 3.6)*†
TS^WUR^ (ratio)	1.7 (1.2–2.2)*	1.7 (1.3–2.0)	2.0 (1.3 to 5.1)	2.0 (1.5–4.5)*	2.9 (2.1 to 4.5)
CPM^PPT-mean^ (kPa)	76.2 (7.9–204.9)	117.6 (53.6–167.4)	67.3 (22.3 to 159.3)	133.9 (54.5–202.7)	93.1 (36.8 to 193.7)
CPM^Unc^ (kPa)	122.0 (26.3–219.5)†	107.3 (56.1–178.7)	74.0 (−27.6 to 106.1)†	103.9 (53.7–208.8)	95.6 (−2.0 to 211.2)

	Baseline			Follow-up	
	Healthy^forearm^		LBP^forearm^	Healthy^forearm^	LBP^forearm^
	Rater 3	Rater 2	Rater 3	Rater 3	Rater 3
Forearm					
PPT (kPa)	222.0 (176.9–249.5)	206.3 (147.0–275.4)	271.5 (195.5 to 305.3)	224.0 (178.4–251.9)	216.5 (164.6–281.6)
TS^WUD^ (−10 to 10)	1.2 (0.5–2.2)	1.4 (0.5–2.2)	1.5 (0.5 to 2.5)	0.9 (0.3–2.0)	1.3 (0.4–2.3)
TS^WUR^ (ratio)	2.5 (1.9–3.8)†‡	3.6 (2.0–5.4)‡	5.0 (2.3 to 9.5)†	2.6 (1.7–4.6)	3.5 (2.1–7.5)
CPM^PPT-mean^ (kPa)	87.2 (50.4–119.9)	109.3 (42.1–173.0)	55.2 (24.2 to 91.8)	66.6 (36.9–131.0)	62.7 (31.0–99.3)
CPM^Unc^ (kPa)	92.1 (37.2–163.6)	120.5 (30.3–213.6)	47.0 (−6.9 to 98.0)	55.9 (5.9–95.0)	38.2 (11.8–81.4)

Data are presented as median (IQR).

* Paired samples *t* test (normal) or Wilcoxon signed rank test (nonnormal) demonstrating significant difference between baseline and follow-up measurements (*P* < 0.05).

† Independent samples *t* test (normal) or Mann–Whitney *U* test (nonnormal) demonstrating significant differences in QST modalities between healthy and diseased participants (*P* < 0.05).

‡ Paired samples *t* test (normal) or Wilcoxon signed rank test (nonnormal) demonstrating significant difference between baseline measurements from rater 1 or 3, with rater 2 in healthy participants (*P* < 0.05).

CPM^PPT-mean^, conditioned pain modulation where the mean of the 3 PPT measurements was used as an unconditioned stimulus; CPM^Unc^, conditioned pain modulation where a unique PPT measurement was used as an unconditioned stimulus; LBP, participants with low back pain; PPT, mean pressure pain threshold; RA, participants with rheumatoid arthritis; TS^WUD^, temporal summation calculated as a difference; TS^WUR^, temporal summation calculated as a ratio.

**Table 3:** Interrater and test–retest reliability in all participants.

	Lower leg		
	Healthy^leg^		RA^leg^
	Interrater (rater 1 − rater 2) (n = 20)	Test–retest (rater 1) (n = 20)	Test–retest (rater 1) (n = 18)
	ICC (95% CI)	ICC (95% CI)	ICC (95% CI)
PPT	0.92 (0.82, 0.97)	0.95 (0.88, 0.98)	0.94 (0.84, 0.98)
TS^WUD^	0.95 (0.87, 0.98)	0.86 (0.68, 0.94)	0.77 (0.39, 0.92)
TS^WUR^	0.61 (0.26, 0.82)	0.68 (0.33, 0.86)	0.56 (0.13, 0.81)
CPM^PPT-mean^	0.01 (−0.45, 0.46)	0.64 (0.30, 0.84)	0.11 (−0.34, 0.53)
CPM^Unc^	0.19 (−0.29, 0.58)	0.71 (0.39, 0.87)	−0.02 (−0.40, 0.41)

	Forearm		
	Healthy^forearm^		LBP^forearm^
	Interrater (rater 3 − rater 2) (n = 25)	Test–retest (rater 3) (n = 25)	Test–retest (rater 3) (n = 25)
	ICC (95% CI)	ICC (95% CI)	ICC (95% CI)
PPT	0.86 (0.72, 0.94)	0.77 (0.54, 0.89)	0.92 (0.83, 0.96)
TS^WUD^	0.88 (0.74, 0.94)	0.76 (0.52, 0.89)	0.78 (0.56, 0.90)
TS^WUR^	0.72 (0.41, 0.87)	0.48 (0.11, 0.73)	0.71 (0.45, 0.86)
CPM^PPT-mean^	0.46 (0.09, 0.72)	0.43 (0.06, 0.70)	0.44 (0.07, 0.71)
CPM^Unc^	0.55 (0.21, 0.77)	0.50 (0.15, 0.74)	−0.10 (−0.44, 0.27)

Intraclass correlation coefficient (ICC) with 95% confidence intervals (CI) are presented.

CPM^PPT-mean^, conditioned pain modulation where the mean of the 3 PPT measurements was used as an unconditioned stimulus; CPM^Unc^, conditioned pain modulation where a unique PPT measurement was used as an unconditioned stimulus; LBP, participants with low back pain; PPT, pressure pain threshold; RA, participants with rheumatoid arthritis; TS^WUD^, temporal summation calculated as a difference (logarithmic transformed); TS^WUR^, temporal summation calculated as a ratio (logarithmic transformed).

Baseline TS^WUD^ measurements were statistically similar between assessments, although RA^leg^ showed a change over time (*z* = −2.32, *P* = 0.02, Table 2). Intraclass correlation coefficients for interrater and test–retest ranged from 0.76 to 0.95, displaying high to very high reliability at the lower leg and a high reliability at the forearm (Table 3). Bland–Altman plots (Figs. 1c, d, 2c, d, 3c, d, Supplementary Table 1, available at http://links.lww.com/PR9/A208) did not show wide limits of agreement. The ICCs for TS^WUD^ were statistically similar between lower leg and forearm. Measurements of wind-up ratio (TS^WUR^) showed differences between raters (Healthy^forearm^ at baseline; median rater 3 = 2.5, rater 2 = 3.6, *z* = −2.46, *P* = 0.01) and between baseline and follow-up (Healthy^leg^ median baseline = 1.7, follow-up = 2.0, *z* = −2.27, *P* = 0.02, Table 2). Reliabilities of TS^WUR^ were classified as comparatively lower in some study populations and test sites. Healthy^leg^, Healthy^forearm^, RA, and LBP participants' test–retest ICCs showed low-to-moderate reliability (ICC = 0.48–0.72) (Table 3). Interrater reliability for Healthy^leg^ was similar to Healthy^forearm^. Bland–Altman plots showed greater variability at larger values of TS^WUR^, particularly in disease populations (Supplementary Figure 1a–1f, available at http://links.lww.com/PR9/A208).

Baseline CPM^PPT-mean^ showed no significant differences in measurements between assessments (Table 2). The ICCs for CPM^PPT-mean^ were heterogeneous with values between 0.01 and 0.64, classified as no to moderate reliability. For Bland–Altman plots, LoA between measurements from raters were generally wide (Figs. 1e, f, 2e, f, 3e, f and Supplementary Table 1, available at http://links.lww.com/PR9/A208). No differences were found for ICCs for CPM^PPT-Mean^ between the lower leg and forearm. Baseline CPM^Unc^ showed statistically similar measurements between raters and in test–retest reliability (Table 2) but also displayed heterogeneous ICC values in healthy adults at both lower leg and forearm (ICC = 0.19–0.71) (Table 3). The CPM^Unc^ measures also showed no test–retest reliability in either RA or LBP (ICC = −0.02 and −0.10, respectively) (Table 3; Supplementary Figure 2a-2f, available at http://links.lww.com/PR9/A208). No differences were found for ICCs of CPM^Unc^ between the lower leg and forearm.

Correlations between modalities demonstrated that a higher PPT was associated with a lower TS^WUD^ in people with RA and LBP, a higher CPM^Unc^ in all participant groups, and higher CPM^PPT-mean^ in Healthy^forearm^ participants (Supplementary Table 2, available at http://links.lww.com/PR9/A208). TS^WUD^ also displayed correlations with PPT^mean^ and CPM^PPT-mean^ in some populations (Supplementary Table 2, available at http://links.lww.com/PR9/A208). Participants' age was not significantly correlated with QST outcomes for most modalities (Supplementary Table 3, available at http://links.lww.com/PR9/A208). LBP^forearm^ participants had a higher rater 3 baseline TS^WUR^ than Healthy^forearm^ participants (Mann–Whitney *U* = 200.00, *P* = 0.03). In addition, when compared with Healthy^leg^ participants, RA^leg^ participants had lower rater 1 baseline CPM^Unc^ (t = 2.35, *P* = 0.02) and higher follow-up TS^WUD^ (Mann–Whitney *U* = 110.50, *P* = 0.04) (Table 2). Lower PPT was reported by female participants for all rater 1 comparisons at the tibialis anterior (lower leg) and at baseline for rater 3 at the brachioradialis (forearm) (Supplementary Table 4, available at http://links.lww.com/PR9/A208).

## 4. Discussion

This study found that PPT and TS were reliable modalities to measure aspects of central pain processing. These modalities seem to be transferable between diagnoses as disparate as LBP and RA. Additionally, they are transferable between different body sites that are distant from the index sites of pain, and their correlations are consistent with QST measuring underlying central sensitisation.

Pain detection threshold was the most consistently reliable QST modality across populations, time-points, and raters, with no obvious systematic patterns of heterogeneity. This study extends previous studies that have demonstrated high reliability (ICC = 0.75–0.94) of PPT in healthy participants,^8,9,11,42,45^ across different time intervals (10 minutes to 6 hours),^8,48^ and in people with knee osteoarthritis or neuropathic pain,^14,61^ RA, or LBP.^29,46^ Both the brachioradialis and the tibialis anterior can be recommended as sites for PPT. Our Bland–Altman plots illustrated little systematic variability between PPT measurements in all groups of participants. The absolute variability of the data in some individuals between tests may extend beyond the minimum clinically important difference (MCID), if defined using the common derivation of 0.5 SD.^40^ However, it is not currently known how strongly pain mechanisms map onto patient-reported outcomes such as pain, and the clinical importance of differences for QST remains uncertain. Our data extend previous findings of PPT reliability^61^ to show similar results in healthy, RA, and LBP participants, with PPT conducted at different body sites. High reliability of PPT might therefore be a transferable and generalisable finding. Our study used a longer gap between test–retest sessions than previous studies,^55^ and our very high level of test–retest reliability over 1 to 3 weeks suggests that pain pressure sensitivity is a highly stable trait.^41^

Conceptually, TS may describe the excitability of spinal cord neurons as it plateaus after frequent stimulation^51^ and can be routinely used in clinics.^52,61^ This study found that TS^WUD^ showed high to very high reliability, with no obvious systematic patterns of heterogeneity. Our findings are consistent with previous evidence of TS test–retest reliability in healthy participants (ICC = 0.67–0.87)^7,19,27^ and patients.^2^ Although some studies have shown low test–retest reliability (ICC = 0.43) and interrater reliability (ICC = 0.41),^49^ our data support the idea that TS tests could be similarly reliable between sites or diagnostic groups.

We explored calculation methods for TS. Temporal summation is often calculated as a ratio (TS^WUR^)^51,52^ comparable with calculation of wind-up ratio in electrophysiology.^21,51^ However, a precise physiological parallel between TS and electrophysiological wind-up is not proven, and distortion from low denominators could adversely affect statistical properties of TS^WUR^. We found that TS showed consistently high reliability when calculated as a difference between 2 assessments (TS^WUD^). Our data suggest that it could be worth investigating whether there are possible advantages of the TS^WUD^ metric.

Conditioned pain modulation might add important information about descending pain modulation that are not captured by PPT or TS. However, obtaining CPM reliability may be challenging.^26^ We found that test–retest and interrater ICCs for CPM^PPT-mean^ ranged from no to moderate reliability, when calculated with the mean PPT value as an unconditioned stimulus. Conditioned pain modulation can be measured using substantially different methodologies, and careful selection of the best protocol is needed. Our findings are consistent with reported CPM in healthy participants (ICC = 0.60–0.82)^30^ and people with chronic LBP (ICC = 0.59),^36^ shoulder pain (ICC = 0.54),^57^ and chronic pancreatitis (ICC = 0.10).^44^ Several factors might compromise reliability of CPM. The synthesis of multiple measurements and participant self-assessments into a single value might contribute. The underlying mechanisms might be less stable (“more dynamic”), and differences between observations might reflect real changes in descending modulation. The timing and delivery of CPM within a research assessment might be particularly important. Previous studies have shown poor CPM test–retest reliability when a test stimulus has become intolerable.^44^ Future studies might compare CPM reliability between conditioning stimuli of different intensities or modalities. The variability and fluctuating nature of musculoskeletal pain^18^ and the subjective nature of pain perception^61^ may each contribute to low CPM ICCs. The LoA in the Bland–Altman graphs of CPM sometimes seemed to be wider than those of PPT alone, although this was not always the case (eg, CPM at brachioradialis in healthy participants), and the 95% CI for each LoA reveal the degree of uncertainty about the true variation. Variation in the CPM often appeared wider than the 0.5 SD used for MCID calculations,^40^ and therefore, CPM might be the most changeable characteristic or difficult to administer test.

We included CPM as the final modality in our QST protocol to avoid carry-over effects of the conditioning stimulus. Multiple testing with painful stimuli may modulate central pain processing, with increasing sensitivity potentially leading stimuli to approach the pain tolerance threshold. Therefore, forfeiting the interim PPT stimulus might be beneficial. We found CPM reliability was improved if baseline PPT results were taken as the unconditioned values (CPM^PPT-mean^) rather than using a repeated PPT undertaken immediately before application of the conditioning stimulus (CPM^Unc^). When CPM was calculated with an unconditioned stimulus repeated immediately before conditioning (CPM^Unc^), test–retest reliability was negative, indicating no reliability. A previous study has similarly found negative test–retest reliability (ICC = −0.40).^30^ It is possible that the study visit and QST protocol itself activated endogenous pain modulatory pathways, such that PPT immediately before induction of ischaemic pain was already “conditioned.” In summary, CPM^PPT-mean^ demonstrated statistical, methodological, and application advantages.

Central sensitisation results from multiple processes, and different QST modalities might reflect different aspects of central sensitisation rather than each being estimates of a shared “central sensitisation.”^37^ Associations were demonstrated between PPT^mean^ with TS^WUD^, CPM^PPT-mean^, and CPM^Unc^, as well as between TS^WUD^ with CPM^PPT-mean^, suggesting overlapping/interdependent mechanisms. Lower CPM in populations with chronic pain^26,31,43,62^ might indicate deficient endogenous analgesic mechanisms or a lack of reserve within an endogenous inhibitory system that is already fully activated. People with RA and LBP have reduced PPTs, increased TS, and deficient CPM,^35,39^ suggesting changes at multiple levels in pain processing pathways.

Although this study had strengths from use of shared protocols across sites with multiple researchers, it is subject to a number of limitations. Our relatively small sample size increased uncertainty in ICC estimates, with wide CIs failing to rule out lower levels of reliability, even when the point estimate for ICC was within the good to excellent range. When there is low statistical power, it is also possible that statistically nonsignificant results might be because of the sample size. However, our findings are consistent with those from other studies have examined QST reliability with much larger sample sizes.^58,60^ Our study was not designed to detect whether ICC values were different by an amount greater than a clinically important difference. Future work should evaluate what is a clinically important difference in QST measures in relation to important patient-centred outcomes. Our comparisons between ICC values were post hoc, and our sample sizes, comparable with previous reliability studies,^38^ were designed to adequately estimate reliability rather than test hypothesised differences between groups. Some comparisons were not assessed, as interrater reliability was not evaluated in patient participants, to reduce the burden of participants with chronic pain. Test–retest reliability was analogous to intrarater reliability, but additional confounders might have influenced reliability in between sessions. Quantitative sensory testing involves complex procedures influenced by interacting variables, and future research might explore additional mechanisms that underlie observed differences in reliability. Although we studied diverse populations, extension of our findings to other chronic pain diagnoses requires further validation. The different participant groups had different mean ages, which could have influenced the results. The age structure of this study is not representative and should not be used to derive reference QST data or inferences about RA or LBP. However, we believe that the groups with different mean ages may still report reliably. Age might be associated with reporting presence or severity of pain, and future research might explore whether age also can influence the reliability of pain reporting. Comorbidities might also influence reliability if they flare or change severity/activity between sessions. This can be minimised by assessing regions with no reported pain (and verifying this with each participant). We compared reliability between populations and modalities. The Bland–Altman plots also revealed greater variation at higher QST measurement values within a population, and reliability should be measured within any population under study.

To conclude, a QST protocol consisting of PPT and TS, assessed on either the forearm or the leg, is a reliable form of quantifying central pain mechanisms. Further research is needed into the underlying reasons for lower reliability of CPM, possibly in larger samples and different populations.

## Disclosures

D.F.M. has grant support from Eli Lilly and Pfizer for projects outside of this study. D.A.W. has grant support from Eli Lilly, UCB and Pfizer for projects outside of this study. Consultancies for Pfizer, AbbVie, GSK. No other potential conflicts were declared by other authors.

## Supplementary Material

## Supplemental digital content

Supplemental digital content associated with this article can be found online at http://links.lww.com/PR9/A208.
