# Supplementary material for: The interrater and test–retest reliability of 3 modalities of quantitative sensory testing in healthy adults and people with chronic low back pain or rheumatoid arthritis
Source: Pain Rep. 2023 Oct 10;8(6):e1102. doi: 10.1097/PR9.0000000000001102 (PMC10566868; doi:10.1097/PR9.0000000000001102)
Supplement: Supplementary file 1 [file painreports-8-e1102-s001.pdf]

Supplementary Table 1: *Mean differences and limits of agreement for test-retest and inter-rater reliability based on Bland Altman analysis results*

		**Test-retest reliability**			**Inter-rater reliability**		
		Mean difference	-LoA (95% CI)	+LoA (95% CI)	Mean difference	-LoA (95% CI)	+LoA (95% CI)
**Healthy^{leg}**	***PPT***	-34.60	-179.59 (-239.56, -119.63)	110.38 (50.41, 170.34)	25.46	-162.08 (-239.65, -84.52)	213.00 (135.44, 290.57)
	TS^{WUD}	-0.12	-1.53 (-2.11, -0.95)	1.28 (0.70, 1.87)	0.09	-1.19 (-1.72, -0.66)	1.36 (0.83, 1.89)
	TS^{WUR}	1.08	-6.25 (-9.28, -3.22)	8.42 (5.38, 11.45)	-1.87	-10.16 (-13.58, -6.72)	6.42 (2.99, 9.84)
	$CPM^{PPT\text{-}mean}$	-34.72	-215.84 (-290.74, -140.93)	146.39 (71.49, 221.30)	-8.37	-330.46 (-463.67, -197.25)	313.73 (180.52, 446.94)
	CPM^{Unc}	-4.23	-184.82 (-259.51, -110.13)	176.36 (101.67, 251.05)	2.34	-299.38 (-424.16, -174.59)	304.06 (179.27, 428.84)
**Healthy^{forearm}**	***PPT***	-7.53	-233.85 (-316.41, -151.30)	218.79 (136.23, 301.34)	-4.19	-217.34 (-295.10, -139.59)	208.97 (131.22, 286.72)
	TS^{WUD}	0.15	-1.23 (-1.73, -0.73)	1.52 (1.02, 2.02)	-0.17	-1.47 (-1.94, -1.00)	1.12 (0.65, 1.60)
	TS^{WUR}	-1.36	-9.33 (-12.24, -6.43)	6.60 (3.69, 9.50)	-0.85	-6.22 (-8.18, -4.26)	4.52 (2.56, 6.48)
	$CPM^{PPT\text{-}mean}$	-10.06	-217.55 (-293.22, -141.86)	197.42 (121.73, 273.10)	-19.20	-230.14 (-307.09, -153.20)	191.74 (114.80, 268.69)
	CPM^{Unc}	19.59	-199.75 (-279.76, -119.74)	238.94 (158.93, 318.95)	-44.34	-251.03 (-326.42, -175.64)	162.34 (86.95, 237.74)
**RA^{leg}**	***PPT***	-14.10	-189.15 (-267.23, -112.68)	161.74 (84.47, 239.02)	[redacted]	[redacted]	[redacted]
	TS^{WUD}	-0.74	-3.02 (-4.02, -2.02)	1.53 (0.53, 2.53)	[redacted]	[redacted]	[redacted]
	TS^{WUR}	1.90	14.83 (-22.17, -7.48)	18.62 (11.27, 25.97)	[redacted]	[redacted]	[redacted]
	$CPM^{PPT\text{-}mean}$	-42.65	-325.39 (-449.64, -201.14)	240.09 (115.84, 364.33)	[redacted]	[redacted]	[redacted]
	CPM^{Unc}	-66.34	-368.51 (-501.29, -235.72)	235.82 (103.03, 368.61)	[redacted]	[redacted]	[redacted]
**LBP^{forearm}**	***PPT***	18.13	-83.55 (-120.64, -46.46)	119.81 (82.72, 156.90)	[redacted]	[redacted]	[redacted]
	TS^{WUD}	0.01	-1.60 (-2.18, -1.01)	1.61 (1.03, 2.20)	[redacted]	[redacted]	[redacted]
	TS^{WUR}	2.61	-12.00 (-17.33, -6.67)	17.22 (11.89, 22.55)	[redacted]	[redacted]	[redacted]
	$CPM^{PPT\text{-}mean}$	-19.26	-192.28 (-255.39, -129.16)	153.77 (90.65, 216.88)	[redacted]	[redacted]	[redacted]
	CPM^{Unc}	-16.89	-271.24 (-364.02, -178.46)	237.46 (144.68, 330.24)	[redacted]	[redacted]	[redacted]

Note: LoA= limit of agreement, CI= confidence interval, PPT: Pressure Pain Threshold, TS^{WUD}: Temporal Summation calculated as a difference, TS^{WUR}: Temporal Summation calculated as a ratio $CPM^{PPT\text{-}mean}$: Conditioned Pain Modulation where the mean of the three PPT measurements was used as an unconditioned stimulus, CPM^{Unc}: Conditioned Pain Modulation where a unique PPT measurement was used as an unconditioned stimulus, RA= participants with Rheumatoid Arthritis, LBP= participants with low back pain.

Supplementary Table 2: *Correlations between QST modalities in different participant groups*

	**Healthy^{leg}:** Rater 1		**Healthy^{leg}:** Rater 2	**RA^{leg}:** Rater 1		**Healthy^{forearm}:** Rater 3		**Healthy^{forearm}:** Rater 2	**LBP^{forearm}:** Rater 3	
	Baseline	Follow-up	Baseline	Baseline	Follow-up	Baseline	Follow-up	Baseline	Baseline	Follow-up
PPT^{mean} – TS^{WUD}	-0.38 (0.10)	-0.29 (0.22)	-0.33 (0.16)	-0.61 (<0.01)*	-0.44 (0.07)	0.02 (0.91)	-0.35 (0.09)	-0.03 (0.87)	-0.42 (0.04)*	-0.60 (<0.01)*
PPT^{mean} – TS^{WUR}	-0.36 (0.12)	0.03 (0.91)	-0.29 (0.22)	-0.26 (0.30)	0.16 (0.54)	-0.14 (0.49)	-0.18 (0.38)	0.08 (0.75)	0.11 (0.62)	-0.03 (0.90)
TS^{WUD} – CPM$^{PPT\text{-}mean}$	-0.30 (0.21)	0.02 (0.94)	0.03 (0.91)	-0.26 (0.30)	-0.04 (0.86)	-0.14 (0.51)	-0.19 (0.35)	-0.40 (<0.05)*	0.13 (0.53)	-0.24 (0.24)
PPT^{mean} – CPM$^{PPT\text{-}mean}$	0.02 (0.94)	-0.04 (0.86)	0.29 (0.21)	-0.01 (0.98)	0.40 (0.10)	<-0.01 (0.99)	0.19 (0.36)	0.39 (<0.05)*	0.26 (0.21)	0.26 (0.22)
PPT^{mean} – CPM^{Unc}	0.11 (0.64)	-0.07 (0.78)	0.62 (<0.01)*	-0.14 (0.59)	0.59 (0.01)*	0.85 (<0.01)*	0.91 (<0.01)*	0.73 (<0.01)*	0.85 (<0.01)*	0.93 (<0.01)*

Note: data is presented as r (p-value), Spearmans correlation coefficient were conducted for all analyses to allow for comparison. *= p<0.05, showing statistically significant correlation between the two variables.

PPT^{mean} = Mean Pressure-Pain Threshold, TS^{WUD}= Temporal Summation calculated as a difference (logarithmically transformed), TS^{WUR}: Temporal Summation calculated as a ratio (logarithmically transformed), CPM$^{PPT\text{-}mean}$: Conditioned Pain Modulation where the mean of the three PPT measurements was used as an unconditioned stimulus, CPM^{Unc}: Conditioned Pain Modulation where a unique PPT measurement was used as an unconditioned stimulus, RA= participants with Rheumatoid Arthritis, LBP= participants with low back pain, Rater 1= Sophia Brady, Rater 2= Daniel McWilliams, Rater 3= Vasileios Georgopoulos.

Supplementary Table 3: *Correlations between QST modalities with age in pooled participant groups*

	Healthy^{leg} and RA^{leg} participants			Healthy^{forearm} and LBP^{forearm} participants		
	Rater 1 (n=38)		Rater 2 (n=20)	Rater 3 (n=50)		Rater 2 (n=25)
	Baseline	Follow-up	Baseline	Baseline	Follow-up	Baseline
PPT	-0.19 (0.27)	-0.27 (0.10)	-0.28 (0.23)	0.01 (0.97)	-0.02 (0.89)	-0.10 (0.65)
TS^{WUD}	0.24 (0.14)	0.40 (0.01)*	0.39 (0.09)	0.08 (0.60)	0.10 (0.51)	-0.18 (0.38)
CPM$^{PPT-mean}$	-0.07 (0.67)	-0.12 (0.48)	-0.40 (0.08)	-0.20 (0.17)	0.02 (0.88)	-0.10 (0.63)

Note: data is presented as r (p-value), Spearmans correlation coefficient were conducted for all analyses to allow for comparison. *= p<0.05, showing statistically significant correlation between the two variables.
PPT = Mean Pressure-Pain Threshold, TS^{WUD}= Temporal Summation calculated as a difference, CPM$^{PPT-mean}$ = Conditioned Pain Modulation where the mean of the three PPT measurements was used as an unconditioned stimulus, RA= participants with Rheumatoid Arthritis, LBP= participants with low back pain.

Supplementary Table 4: *Sex differences between QST modalities in pooled participant groups*

	**Healthy^{leg} and RA^{leg} participants**						**Healthy^{forearm} and LBP^{forearm} participants**					
	Rater 1 (n=38)				Rater 2 (n=20)		Rater 3 (n=50)				Rater 2 (n=25)	
	Baseline		Follow-up		Baseline		Baseline		Follow-up		Baseline	
	Male	Female	Male	Female	Male	Female	Male	Female	Male	Female	Male	Female
PPT	605.87 (204.93)*	328.79 (200.92)*	655.99 (244.32)*	337.24 (212.41)*	558.38 (216.42)	362.14 (225.44)	339.85 (159.85)*	217.37 (84.76)*	320.81 (209.20)	219.80 (84.90)	373.92 (292.56)	191.10 (84.95)
TS^{WUD}	0.95 (0.63)	1.81 (1.57)	1.30 (0.76)	2.27 (1.58)	1.13 (0.56)	1.24 (1.12)	1.73 (1.42)	1.44 (1.34)	1.61 (1.44)	1.38 (1.44)	2.00 (1.47)	1.50 (1.68)
CPM$^{PPT-mean}$	82.47 (127.13)	95.20 (96.45)	137.30 (115.53)	123.02 (107.81)	149.71 (94.44)	62.00 (102.02)	63.90 (116.36)	76.94 (45.26)	92.10 (80.70)	83.99 (98.46)	64.46 (126.22)	131.94 (108.37)

Note: data are presented as means (standard deviations). *= $p<0.05$, showing statistically significant sex difference in measurements calculated using independent samples t-tests (normal data) and Mann-Whitney U tests (non-normal data).

PPT = Mean Pressure-Pain Threshold, TS^{WUD}= Temporal Summation calculated as a difference, CPM$^{PPT-mean}$ = Conditioned Pain Modulation where the mean of the three PPT measurements was used as an unconditioned stimulus, RA= participants with Rheumatoid Arthritis, LBP= participants with low back pain.

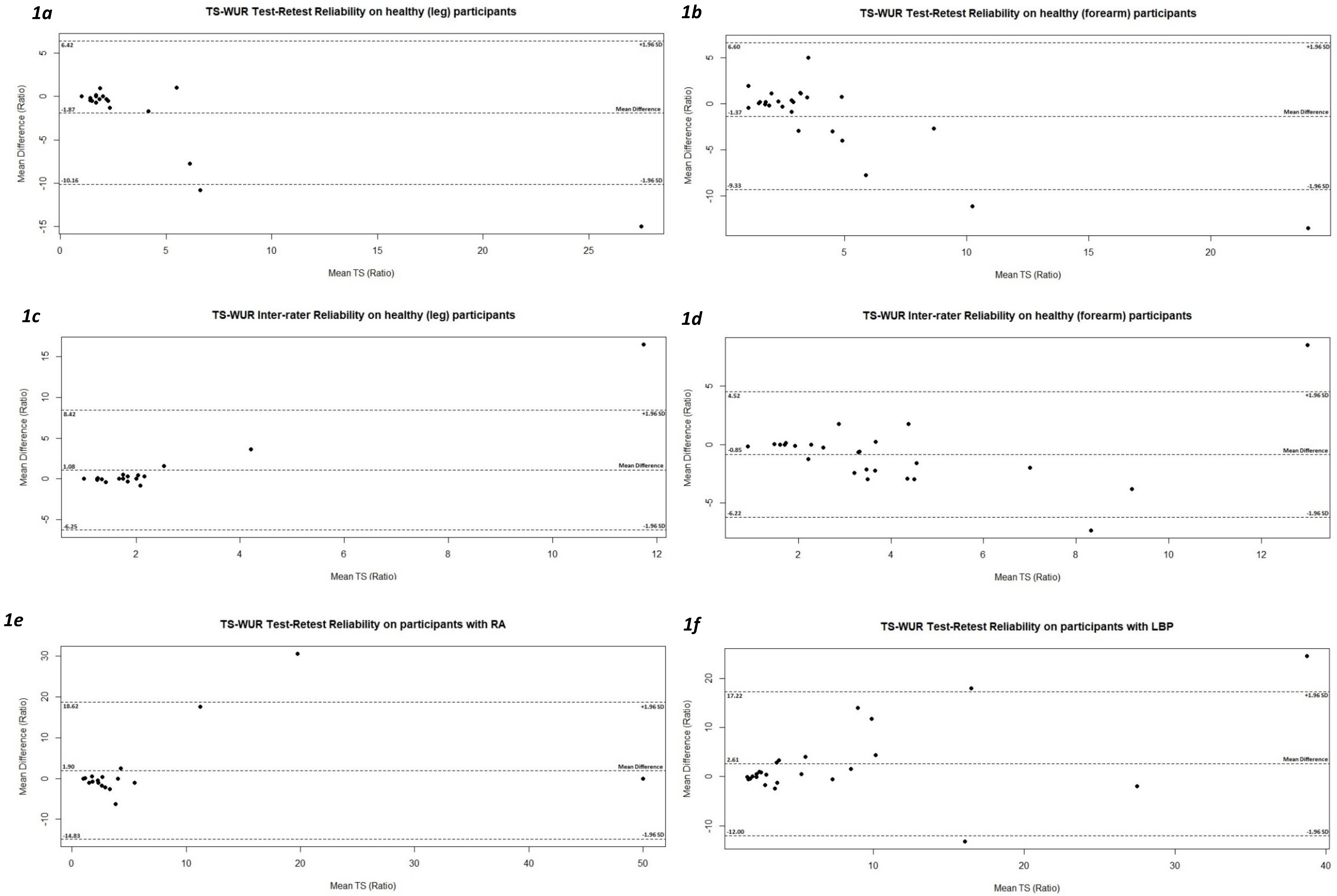

Supplementary Figure 1a-1f: *Alternate TS calculation method (TS^{WUR}) across populations and raters*

Note: LoA= limit of agreement, TS^{WUR}= Temporal Summation calculated as a ratio, RA= participants with Rheumatoid Arthritis, LBP= participants with low back pain

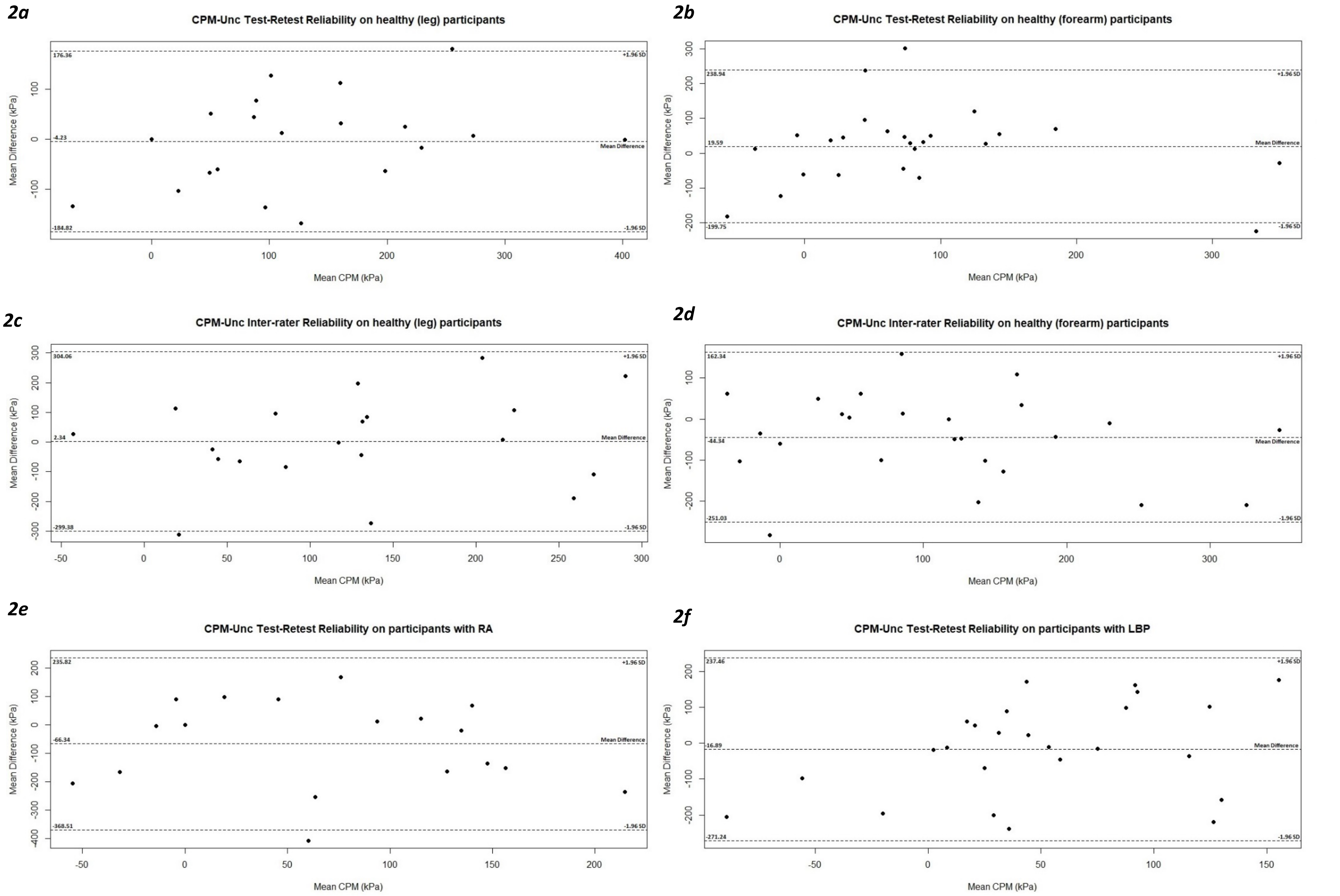

Supplementary Figure 2a-2f: *Alternate CPM calculation method (CPM^{Unc}) across populations and raters*

Note: LoA= limit of agreement, CPM^{Unc}= Conditioned Pain Modulation where a unique PPT measurement was used as an unconditioned stimulus, RA= participants with Rheumatoid Arthritis, LBP= participants with low back pain
